# Solitary Laryngeal Metastasis from Transitional Cell Carcinoma of the Kidney: Clinical Case and Review of the Literature

**DOI:** 10.1155/2015/595283

**Published:** 2015-07-06

**Authors:** Tarek Assi, Elie El Rassy, Amine Haddad, Joseph Kattan

**Affiliations:** ^1^Hematology-Oncology Department, Faculty of Medicine, Saint Joseph University, Beirut 1104 2020, Lebanon; ^2^Otorhinolaryngology Department, Faculty of Medicine, Saint Joseph University, Beirut 1104 2020, Lebanon

## Abstract

The urogenital tract is a rare origin of laryngeal metastasis; transitional cell carcinoma with laryngeal metastases had never been reported previously. In this paper, we describe the clinical and pathological characteristics, evolution, and treatment of the first reported case of a laryngeal metastasis of a TCC followed by a brief review of the literature.

## 1. Background

Transitional cell carcinoma (TCC) of the renal pelvis accounts for approximately 5–7% of all urothelial tumors [1]. It usually presents with local disease but may be metastatic in up to 5% of patients affecting most commonly the liver, the lungs, and bones [2]. The literature describes several unusual metastatic locations including the clitoris and the duodenum but has never reported laryngeal metastasis [3]. In this paper, we describe the clinical and pathological characteristics, evolution, and treatment of the first reported case of a laryngeal metastasis of a TCC.

## 2. Case History

A 70-year-old male patient was referred to our clinic for a new onset of hematuria, fatigue, and weight loss. Clinical exam was strictly normal. Contrast enhanced abdominal and pelvic CT scan demonstrated a renal mass classified Bosniak V. Total left nephrectomy revealed a necrotic mass determined as stage III TCC (T3N0M0, G3). Subsequently, the patient received adjuvant chemotherapy of Gemcitabine and Cisplatin. One month later, the patient reported a progressive onset of low grade dysphonia and dysphagia, without signs of systemic symptoms. Clinical exam revealed a solid and painless cervical mass in contact with the right thyroid lobe. Initial laboratory testing showed no abnormalities. Cervical CT scan demonstrated an erosion of the right thyroid cartilage at the level of the glottis and the supraglottic regions attributed to a mass arising from the right lateral laryngeal wall. This lesion was solely metabolically hyperactive (SUV = 6.6) on PET-CT scan. Transcutaneous Trucut biopsy revealed a well differentiated TCC with prominent pleomorphic nuclei on hematoxylin and eosin stain. Immunohistochemistry was diffusely positive for CK 5/6/7, thus closely resembling the bladder tumor pattern of our patient. Salvage chemotherapy with vinflunine was initiated. Unfortunately, the disease progressed with aggravation of the dyspnea until the patient's death three weeks later.

## 3. Discussion

Tumors of the urogenital tract are a rare origin of laryngeal metastasis but may occur even years after complete remission. This is attributed to the high metastatic potential of this tumor in the presence of a highly vascularized tissue. The literature describes rare cases of renal cell carcinoma metastasizing to the larynx but reports of TCC with laryngeal metastasis are inexistent [4]. Laryngeal metastasis is a rare disease that accounts for 0.09–0.4% of all laryngeal tumors and only occurs in advanced stages of primary tumors. It affects most commonly the supraglottis and less frequently the glottis [5]. Symptoms are most often equivocal to symptoms of primary laryngeal tumors. It is noteworthy that the presence of other symptomatic organ dysfunctions and the presence of hemoptysis are possible clues of a metastatic disease [6].

Metastasis to the larynx is determined by its anatomic characteristics. Effectively, the anatomic distribution of the vascular and lymphatic circulation accounts for the rare occurrence of laryngeal seeding and renders the supraglottic and subglottic region the most common sites of metastases [5]. Described metastatic patterns include orderly cascade or retrograde seeding from melanomas and carcinomas of the kidney, breast, lung, prostate, and colon [5, 6]. In the particular case of a primary kidney tumor, as in our patient, the vertebral and epidural veins play a major role in the dissemination of cancer cells and determine the metastatic pattern. These veins are not equipped with valves; thus increased intra-abdominal pressure creates a retrograde flux that disseminates cancer cells to the head and neck region [7]. In the absence of liver and lung metastasis, the main metastatic pattern of our patient involves migration of the tumor cells through Batson plexus or lymphatic canal into the arterioles of the larynx.

Treatment of metastatic kidney cancers relies mainly on the site of metastasis and the performance status of the patient [7]. Whereas surgery is considered only in the palliative setting, systemic therapy is the mainstay of metastatic disease. The comorbidities of our patient and his poor performance status did not allow a surgical approach despite the persistence of dyspnea. Unfortunately, the disease did not reply to treatment and progressed till death three months later.

To our knowledge, this is the first case of laryngeal metastasis from TCC of the renal pelvis to be reported in the literature. The particularity of this case resides in the presence of a solitary laryngeal metastasis where such a finding may be mistaken for a primary tumor of the larynx. The core tip of this case is to maintain a high index of suspicion for laryngeal metastasis in patients with laryngeal symptoms and a past history of cancer. Early detection allows appropriate treatment of this unusual metastatic site.
